# Modified MLVA for Genotyping Queensland Invasive *Streptococcus pneumoniae*

**DOI:** 10.1371/journal.pone.0121870

**Published:** 2015-04-29

**Authors:** Rachael E. Rayner, John Savill, Louise M. Hafner, Flavia Huygens

**Affiliations:** 1 School of Biomedical Sciences, Queensland University of Technology, Brisbane, Australia; 2 Institute of Health and Biomedical Innovation, Queensland University of Technology, Brisbane, Australia; 3 Public Health Microbiology Laboratory, Queensland Health Forensic and Scientific Services, Brisbane, Australia; Naval Research Laboratory, UNITED STATES

## Abstract

**Background:**

Globally, over 800 000 children under five die each year from infectious diseases caused by *Streptococcus pneumoniae*. To understand genetic relatedness between isolates, study transmission routes, assess the impact of human interventions e.g. vaccines, and determine infection sources, genotyping methods are required. The ‘gold standard’ genotyping method, Multi-Locus Sequence Typing (MLST), is useful for long-term and global studies. Another genotyping method, Multi-Locus Variable Number of Tandem Repeat Analysis (MLVA), has emerged as a more discriminatory, inexpensive and faster technique; however there is no universally accepted method and it is currently suitable for short-term and localised epidemiology studies. Currently Australia has no national MLST database, nor has it adopted any MLVA method for short-term or localised studies. This study aims to improve *S*. *pneumoniae* genotyping methods by modifying the existing MLVA techniques to be more discriminatory, faster, cheaper and technically less demanding than previously published MLVA methods and MLST.

**Methods:**

Four different MLVA protocols, including a modified method, were applied to 317 isolates of serotyped invasive *S*. *pneumoniae* isolated from sterile body sites of Queensland children under 15 years from 2007–2012. MLST was applied to 202 isolates for comparison.

**Results:**

The modified MLVA4 is significantly more discriminatory than the ‘gold standard’ MLST method. MLVA4 has similar discrimination compared to other MLVA techniques in this study). The failure to amplify particular loci in previous MLVA methods were minimised in MLVA4. Failure to amplify BOX-13 and Spneu19 were found to be serotype specific.

**Conclusion:**

We have modified a highly discriminatory MLVA technique for genotyping Queensland invasive *S*. *pneumoniae*. MLVA4 has the ability to enhance our understanding of the pneumococcal epidemiology and the changing genetics of the pneumococcus in localised and short-term studies.

## Introduction

More than 800 000 children under five succumb to invasive pneumococcal diseases (IPD) each year globally [1]. Australia is not exempt from invasion with 6.7 per 100 000 notifications in 2013 [2–3]. IPD are defined as the isolation of *S*. *pneumoniae* from normally sterile body sites including blood, tissues, and cerebrospinal, joint, pericardial or pleural fluids [4]. The changing pneumococcal population structure worldwide is largely the result of serotype replacement and capsule switching, especially in first world countries such as Australia where vaccines are widely implemented [5–7]. Serotype replacement has been problematic as current pneumococcal vaccines only target 13 out of 95 pneumococcal serotypes [8–13]. Capsule switching, the transfer of a capsule genes from one pneumococcus to another, is a regular occurrence in pneumococcal populations however it has the potential to reduce vaccine efficacy because vaccine escape isolates can emerge [6–7]. Vaccine escape isolates can develop within 2–3 years of a vaccine introduction, as already detected in the USA and Italy [6, 14]. *In vitro* studies have demonstrated that capsule switching can also impact pneumococcal virulence properties, particularly since the polysaccharide capsule is a virulence factor of pneumococci [8]. A highly virulent capsule type 5 strain was rendered avirulent when expressing a capsule type 3 and a type 6A strain expressing a capsule type 6C was more virulent than wild types [15–16].

So far, there have been little published studies examining the pneumococcal population structure in Australia since the introduction of the new vaccine 13-valent pneumococcal conjugate vaccine in July 2011 [17].

For decades, the main technique for surveying serotype distribution and replacement has involved serotyping pneumococci with antisera (such as the Quellung reaction) [18]. However serotyping is expensive, laborious, and ambiguous, revealing no information about genetic recombination and capsule switching. Therefore several genotyping methods have been developed, including MLST [19] and several MLVA techniques [20–23]. It is important to bear in mind that genotyping and serotyping are both required in combination to detect capsule switching events.

Commonly, MLST is used to genotype *S*. *pneumoniae* based on the original technique involving housekeeping genes developed by Enright and Spratt [19]. MLST housekeeping genes are considered to be stable and less prone to recombination than the rest of the genome, enabling examination of long-term population changes and studies across wider areas [19]. However, MLST is expensive and laborious, not suitable for large-scale genotyping or routine use [20–21, 24–27]. There is no national Australian MLST database, and the international MLST database only contains 138 Australian isolates from the year 1967 to 2013, providing very little information regarding the Australian pneumococcal population. The international MLST database could be used as a comparison against the pneumococcal isolates found in the rest of the world.

To reduce cost and labour intensity, MLVA was developed for genotyping *S*. *pneumoniae*. It is reported to be more discriminatory, inexpensive and faster than MLST, however more suitable for short-term epidemiology changes and localised outbreaks [20–21, 27]. MLVA involves amplifying Variable Number of Tandem Repeats (VNTR) loci and sizing the PCR products according to fragment lengths. VNTRs are suitable genotyping targets since there are multiple loci throughout the genome and they are polymorphic [28]. MLVA can be multiplexed, and uses a DNA sequencer for high throughput analysis of fragment sizes, not sequencing [25].

Different MLVA protocols exist including Koeck’s *et al*. [21] MLVA which amplifies seventeen VNTRs, each in a singleplex PCR. This method was used to genotype pneumococcal isolates in Burkina Faso [29]. Unfortunately the practicality of typing seventeen targets and the difficulty comparing analysis of gels are some of the limitations. There is a freely accessible database (http://mlva.u-psud.fr/mlvav4/genotyping/index.php - Simple Databases labelled ‘Streptococcus pneumoniae2005) which allows comparison of profiles, however there are only 59 isolates available in this database. Recently this protocol has been modified by Van Cuyck *et al*. [22] by reducing the number of VNTRs to seven, which were experimentally determined to have the highest discrimination (Hunter-Gaston Diversity >0.8) within a population of 331 UK isolates. This seven marker MLVA is claimed to be a minimum universal set, ideal for genotyping pneumococcal isolates. However it is known that pneumococcal populations differ between countries, therefore the selection of these seven MLVA markers may not be suitable for Australian isolates.

Another MLVA protocol was developed by Elberse *et al*. [20], which utilises eight BOX VNTR loci amplified in two multiplex PCRs with fluorescently labelled probes. BOX loci are a type of VNTR loci, distinguishable by varying numbers of *boxB* repeat regions (45 base-pair) found between b*oxA* and *boxC* which remain stable under laboratory conditions [20, 30]. BOX elements can form secondary structures and can affect the expression of downstream genes [31]. Elberse’s method solely uses BOX loci (no other VNTR), while Koeck’s method uses a combination of BOX loci and other VNTR loci, which do not contain stable *boxA* and *boxC* units, and vary in repeat lengths e.g. 60 base-pair. Elberse’s MLVA protocol has been applied to genotype pneumococci in the Netherlands and carriage isolates from Portugal, as well as tracking a localised outbreak in England [32]. However, a limitation is that some BOX loci fail to amplify (assigned ‘99’) therefore leaving profiles incomplete, an issue that remains unresolved. An MLVA type can still be assigned even if the profile contains a non-amplified locus (studies from Elberse identify that 89% of serotype 7F isolates will commonly have a BOX-06 that won’t amplify). The limitation of having an incomplete genetic profile is that we can’t see the ‘true’ bacterial fingerprint of the isolate. It is unknown whether this could have implications for population studies.

Finally, Multi-Locus *boxB* Typing (MLBT) was developed, a variation of MLVA by sequencing VNTR loci to detect Single Nucleotide Polymorphisms (SNPs) as well as fragment length variations [23]. MLBT contains VNTRs that have been used in the other MLVA protocols, however the complexities of MLBT does not enable ease of comparison with other MLVA methods [20–21, 23].

The aim of this study was to improve pneumococcal genotyping methods by modifying existing MLVA. It is important to modify the existing MLVA methods to be more discriminatory, faster, cheaper and technically less demanding than previously published MLVA methods and MLST.

## Methods

### Setting

All pneumococcal isolates from Queensland patients with IPD are required to be submitted to the Public Health Microbiology Laboratory at Queensland Health Forensic and Scientific Services (QHFSS), Brisbane. Serotyping (Quellung) is mandatory, however further genotyping has only been performed for research purposes. Invasive *S*. *pneumoniae* isolates taken from normally sterile body sites were serotyped by the Pneumococcal Reference Laboratory, QHFSS, using Quellung reaction [4, 18].

### Laboratory methods

*S*. *pneumoniae* isolates were cultured 16-streak on Horse Blood Agar (HBA, Oxoid, Australia—a commercially available product routinely used for the culture of bacteria). A single colony was re-cultured on fresh HBA to ensure isolates were genetically identical. Isolates were boiled in 400μL TE buffer (~pH8.0) for eight minutes to prepare a thermolysate with template DNA and were stored at -20°C until further use.

MLVA markers were selected from previously published papers [20–23]. All MLVA markers were analysed *in silico* to determine the expected fragment sizes and copy numbers against known *S*. *pneumoniae* genomes from the NCBI database (R6, G54, CGSP14, TIGR4 and Hungary19A-6).

*S*. *pneumoniae* isolates (n = 317) detected in Queensland were genotyped using MLVA1, MLVA2 and MLVA4. Elberse’s MLVA1 contained two multiplexes with eight BOX genes (Table 1) [20]. The MLVA2 procedure was based on Van Cuyck’s MLVA method and contained seven VNTRs (Table 1) [22]. A single multiplex reaction was performed with Spneu17, Spneu19, Spneu27 and Spneu39 as the other three had already been typed in MLVA1. Spneu31 [21] and B10 [23] were separately amplified to determine whether they provided high discrimination within the pneumococcal population, and therefore suitable for the modified MLVA method.

**Table 1:** Multiplex arrangement for MLVA methods.

**Genotyping method**	**Multiplex number**	**Loci**	**Reference**
**MLVA1**	1	BOX-01, BOX-02, BOX-03, BOX-04	[20]
	2	BOX-06, BOX-11, BOX-12, BOX-13	[20]
**MLVA2**	1	Spneu17, Spneu19, Spneu27, Spneu39	[22]
	2	Spneu25, Spneu33, Spneu37	[22]
**MLVA4**	1	BOX-01, B10, Spneu19, Spneu39	This study
	2	BOX-12, BOX-13	This study
	3	BOX-02, BOX-03, BOX-04, Spneu17	This study

MLVA4 was the modified MLVA method based on the high discrimination of the fourteen loci previously used, and the ability to amplify all loci across the different serotypes detected in Queensland [20–23]. Three multiplexes were developed (Table 1). Four primer sets were redesigned due to difficulties in amplification, including BOX-12 (F: GAGATTGCCCTTTTCATCTTCG; R: AGCAACCATTGAAACGCCTG), BOX-13 (F: TCAAAAGATTGGAGAGTTCCGC; R: GGATTTGGAGAGCAAGCAGATC), Spneu19 (F: CACTCACCGTTAGCATTGACTCG; R: TAATCAGGGAGTAGTTGGTTGGG) and B10 (F: GGAGCCGAGTAGGAGATTCTCAC; R: TCGTAGGCTGCTACATTGACCAG) (Geneworks, Australia).

The Corbett Cas1200 Robotic system was used to prepare mastermix with DNA thermolysate. The PCR protocol was optimised and consisted of 15min at 95°C, 30 cycles of 95°C for 30sec, 58°C for 60sec and 72°C for 60sec, followed by extension of 72°C for 10min and held at 4°C. Diluted PCR products (1:150 reverse-osmosis water (PALL, Australia)) were combined with 1200LIZ internal ladder (Applied Biosystems, Australia). A heat denaturation step (95°C for 5min, followed by a hold step at 4°C) was performed on a thermocycler to separate the dsDNA. Fragment sizing was performed on AB3130 (Applied Biosystems, Australia).

Finally, MLST was also applied to selected isolates (n = 202) for comparison purposes as previously described [19, 33].

### Analysis

PeakScanner V1.0 software was used to analyse MLVA results (Applied Biosystems, Australia). A MLVA type (MT) using MLVA1 was assigned to each isolate using the MLVA database (http://www.mlva.net). MT types for MLVA2, and MLVA4 was manually assigned from our own database. Van Cuyck *et al*. [22] did not provide a database or published MLVA types that could be used in the Queensland pneumococcal population. The pneumococcal population structure using all MLVA methods were displayed as eBurst diagrams produced from PHYLOViZ software [34]. Clonal clusters (CC) are defined as two or more isolates that are genetically related and linked by single locus variants (SLV) or double locus variants (DLV). Where an international or larger database is available, Queensland isolates were compared.

MLST results were analysed using ChromasPro software (Technelysium Pty Ltd.) using batch alignment analysis. Allele numbers and sequence types (ST) were assigned to each isolate from the MLST database (www.mlst.net) and displayed as an eBurst diagram using PHYLOViZ software.

The Simpson’s Index of Diversity (S) was calculated to compare the discrimination of all genotyping methods (http://darwin.phyloviz.net/ComparingPartitions/index.php?link=Tool). If the 95% confidence intervals (CI) overlap between methods, the hypothesis that the methods have similar discriminatory power cannot be excluded. The Adjusted Wallace coefficient (AW) was used to measure the congruence between typing methods [35–36].

The frequency of non-amplified loci (‘99’) was compared between each MLVA method to determine whether this was a limitation of a particular method or associated with specific serotypes. Hunter-Gaston Diversity Index (DI) (http://www.hpa-bioinformatics.org.uk/cgi-bin/DICI/DICI.pl) was used to calculate the genetic diversity of VNTR genes within the Queensland population, as used in previous studies [21–22, 37]. DI is a measure of the variation of the number of repeats at each locus, ranging from 0.0 (no diversity) to 1.0 (complete diversity).

### Ethics statement

No human participants were involved directly in this study and hence, human ethics clearance was not required. *S*. *pneumoniae* isolates routinely cultured from clinical specimens were used and we investigated the epidemiology of the *S*. *pneumoniae* isolates, changing genotypes and population structure in Queensland.

## Results

### Comparison of genotyping methods

All 317 *S*. *pneumoniae* Queensland isolates in this study were assigned an MLVA genotype, covering 35 serotypes detected in Queensland from 2007–2012 (serotypes include 1, 3, 4, 6A, 6B, 6C, 7F, 8, 9N, 9V, 10A, 10F, 11A, 12F, 14, 15B, 15C, 16F, 18A, 18B, 18C, 19A, 19F, 22A, 22F, 23A, 23B, 23F, 24F, 33B, 33F, 34, 35B, 35F, and 38). MLVA4 method has novel aspects as four sets of primers and the three multiplex PCR has been newly designed in this study.

Isolates selected for MLST included 13vPCV serotypes and non-13vPCV serotypes to minimise labour work and costs (excluding those originally in the 7vPCV i.e. serotype 4, 6B, 9V, 14, 18C, 19F and 23F, and serotype 19A). Studies have already shown that 7vPCV serotypes have significantly declined [38–39], so we focused on the examination of these recently targeted or non-targeted serotypes.

All three MLVA genotyping methods had a higher discriminatory power compared to MLST (Table 2). MLVA4 had a significantly higher discrimination compared to MLST (S = 0.978 with 106 MLVA types; S = 0.936; 49 ST’s) (n = 202). In comparison, MLVA1 had a discrimination of S = 0.963 (n = 202; 71 MLVA types) and MLVA2 had S = 0.977 (n = 202; 97 MLVA types).

**Table 2:** Calculation of Simpson's Index of Diversity for serotyping (Quellung), MLST, MLVA1, MLVA2 and MLVA4 methods.

***Total Number of S*. *pneumoniae isolates = 202***						
**Molecular method**	**Total number of genotypes**	**Simpson’s Index of Diversity (S)**	**CI (95%)**	**Average time to genotype 48 isolates (days)**	**Cost per isolate (PCR to genotype) (AUS$)**	**Non-amplified loci (%)**
**Serotyping**	29	0.912	0.893–0.930	-	-	N/A
**MLST**	49	0.936	0.920–0.952	16–20	346.65	N/A
**MLVA1**	71	0.963	0.953–0.974	3–4	17.10	20.8
**MLVA2**	97	0.977	0.970–0.985	3–4	16.37	24.3
**MLVA4**	106	0.978	0.971–0.986	3–4	23.71	12.4
***Total Number of S*. *pneumoniae isolates = 317***						
**Serotyping**	35	0.914	0.897–0.932	-	-	N/A
**MLVA1**	163	0.984	0.980–0.989	3–4	17.10	20.2
**MLVA2**	175	0.987	0.983–0.991	3–4	16.37	21.1
**MLVA4**	203	0.990	0.987–0.994	3–4	23.71	12.6

However, when comparing the Adjusted Wallace Coefficient of MLVA4 with the other two MLVA methods, all MLVA methods had similar discriminatory power (Table 2). MLVA4 has high congruence with MLVA1 (AW = 0.883), MLVA2 (AW = 0.766) and MLST (AW = 0.966). This indicates that the MLVA4 genotypes will have a 96.6% probability that it will have the same MLST type. Conversely, the congruency of MLST with MLVA4 (AW = 0.314) indicates that the MLST types will have a 31.4% probability of having the same MLVA4 types, indicating that MLVA4 is more discriminatory.

New MLVA1 BOX alleles were also found including BOX-04 fragment sizes 10 repeats (n = 1), 11 repeats (n = 1) and 12 repeats (n = 3), BOX-03 fragment size 8 repeats (n = 2), BOX-12 fragment size 0 repeats (n = 2) and BOX-13 fragment size >2000bp (unsure how many repeats until sequenced; n = 8). There are 107 new MLVA1 MT types not recorded by the MLVA database [25] In addition, 20 new STs have been submitted to the MLST international database (ID#23211–23429).

### VNTR loci and ‘99’

BOX-01, BOX-04, BOX-12, BOX-13 and Spneu17 had the highest diversity (DI ≥ 0.80) while BOX-02, BOX-11, Spneu19 and Spneu27 had the lowest diversity (DI ≤ 0.66) (Table 3). When examining MLVA1, 20.2% of isolates still contain at least one non-amplified locus. Even MLVA2 contained 21.1% of isolates with non-amplified loci. MLVA4 method reduces the ‘99’ to 12.6% (Table 4).

**Table 3:** Adjusted Wallace Coefficient and 95% confidence intervals (CI) for four genotyping and one serotyping method.

*Total number of S. pneumoniae isolates = 202*					
	MLVA1	MLVA2	MLVA4	Serotyping	MLST
**MLVA1**		0.524 (0.461–0.5287)	0.512 (0.442–0.582)	0.940 (0.891–0.989)	0.984 (0.974–0.995)
**MLVA2**	0.871 (0.800–0.942)		0.739 (0.667–0.810)	0.880 (0.804–0.956)	0.916 (0.887–0.945)
**MLVA4**	0.883 (0.823–0.942)	0.766 (0.690–0.843)		0.918 (0.842–0.994)	0.966 (0.927–1.000)
**Serotyping**	0.372 (0.301–0.442)	0.209 (0.157–0.262)	0.211 (0.161–0.260)		0.648 (0.587–0.709)
**MLST**	0.552 (0.456–0.648)	0.309 (0.244–0.374)	0.314 (0.254–0.374)	0.919 (0.869–0.969)	

**Table 4:** Hunter-Gaston Diversity (DI) for all selected MLVA loci (n = 317 isolates) and frequency of non-amplified loci ‘99’.

MLVA method	Locus	Diversity Index (DI)	CI	Size of tandem repeat (bp)	K	Number of non-amplified loci before singleplex	Number of non-amplified loci after singleplex	Non-amplified serotypes (% of isolates)
**MLVA2, MLVA4**	Spneu17	0.853	0.840–0.866	45	12	6	0	
**MLVA1, MLVA2, MLVA4**	BOX-13	0.821	0.806–0.836	45	10	23	13	33F (5); 6C (7); 19F (1)
**MLVA1, MLVA2, MLVA4**	BOX-12	0.805	0.778–0.833	45	13	89	4	3 (3); 23B (1)
**MLVA1, MLVA4**	BOX-01	0.801	0.785–0.817	45	10	11	4	19F (3), 33B (1)
**MLVA1, MLVA4**	BOX-04	0.797	0.771–0.823	45	12	7	0	
**MLVA2, MLVA4**	Spneu39	0.794	0.775–0.812	45	10	0	0	
**MLVA1, MLVA4**	BOX-03	0.789	0.754–0.824	45	14	28	0	
**MLVA4**	BOX-10	0.784	0.748–0.821	45	11	54	4	15C (2); 19A (2)
	Spneu31	0.729	0.696–0.762	45	11	7	2	
**MLVA1, MLVA2**	BOX-06	0.696	0.653–0.739	45	8	53	32	
**MLVA2, MLVA4**	Spneu19	0.663	0.632–0.694	60	9	24	16	3 (12); 38 (4)
**MLVA1, MLVA4**	BOX-02	0.651	0.628–0.674	45	5	1	0	
**MLVA2**	Spneu27	0.637	0.601–0.674	45	8	13	3	
**MLVA1**	BOX-11	0.392	0.341–0.443	45	3	14	7	
	**TOTAL**	-	-	-	-	330	121	

‘K’ represents the number of different repeats present for each locus in the Queensland pneumococcal sample set.

These VNTR loci failed to amplify in specific serotypes. BOX-06 failed to amplify 75% of serotype 7F (n = 27), BOX-13 failed to amplify 42% of serotype 33F (n = 5) and 54% of serotype 6C (n = 7), Spneu19 failed to amplify 63% of serotype 3 (n = 12) and 50% of serotype 38 (n = 4), and BOX-01 failed to amplify 20% of serotype 19F isolates (n = 3) (Table 3). Spneu19 allele sequences were not found in serotype 3 genomes using BlastN (strains OXC141, SP3, SPN021198, SPN034156, SPN034183, SPN072838, SPN994038 and SPN994039). Similarly Spneu19 failed to amplify in serotype 38; however no serotype 38 genome has been sequenced to date to allow us to determine whether the Spneu19 locus exists. No match was found for BOX-13 in serotype 6C NCBI genomes, and failed to amplify in serotype 33F, however gel electrophoresis showed a large >2000bp fragment (S1 Fig.). BOX-06 primers appear to anneal to a serotype 7F contig (CDC 1087–00 contig ABFT01000005.1) however the fragment size exceeds 1200bp which would not be detected with the AB3130 internal size ladder LIZ1200. Gel electrophoresis did not reveal any bands, corresponding to BOX-06 in serotype 7F.

### Comparison of MLVA and MLST eBurst

MLVA4 has been shown to be vastly cheaper and faster than MLST, costing $23.71 compared to $346.65 per isolate, respectively, and taking only 3–4 days on average to genotype 48 isolates, compared to 16–20 days, respectively.

**Fig 1:**
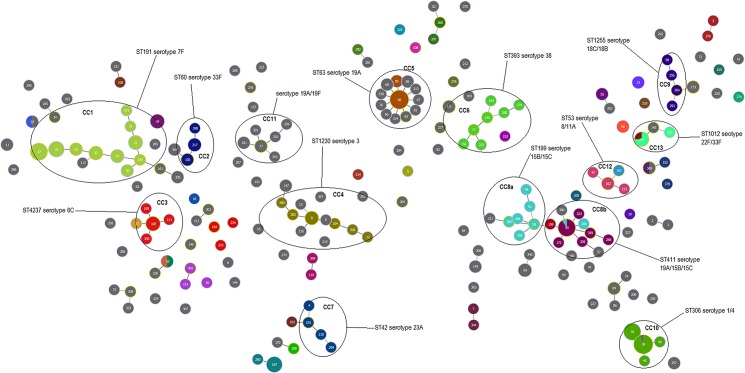
MLVA4 eBurst of Queensland invasive *S*. *pneumoniae* isolates from 2007 to 2012 (n = 317). MLST eBurst of invasive *S*. *pneumoniae* isolates from 2007–2012 (n = 202). MLST results are overlayed (coloured circles) to allow ease of comparison against MLVA4 results. Grey circles represent isolates that have not been assigned a MLST type. The size of the isolate circles corresponds to the number of isolates. Clonal clusters are identified in black ovals, and those that contain predominantly one colour indicate that the isolates have a single MLST type. Isolates are labelled with MLVA4 type (MT).

Comparison of MLST and our MLVA4 eBurst analysis clearly shows two different population structures (Fig. 1). The pneumococcal population structure is displayed according to the MLVA4 genotypes, and it is observed that there are 32 clonal clusters (CC), with the larger eleven clusters labelled in the figure. The MLST results are overlayed on the MLVA4 results; therefore each colour represents a different MLST type. It can be seen that several MLVA4 clusters would appear as a single MLST type, for example CC1 which predominantly contains serotype 7F isolates has as many as ten different MLVA4 types however only one MLST type as depicted by the single green colour of the circles.

Using MLVA4 for localised Queensland studies show that most CCs predominantly contain a single serotype (e.g. CC1 contains serotype 7F, CC4 contains serotype 3) (Fig. 1). However some clusters contain mixed serotypes including CC11 (serotype 19A and 19F), CC8 (serotype 15B, 15C and 19A), CC9 (serotype 18B and 18C), CC10 (serotype 1 and 4), CC3 (serotype 6A and 6C), CC12 (serotype 8 and 11A), CC13 (serotype 33F and 22F). MLST identifies most of these CC but many appear as a single isolates.

**Fig 2:**
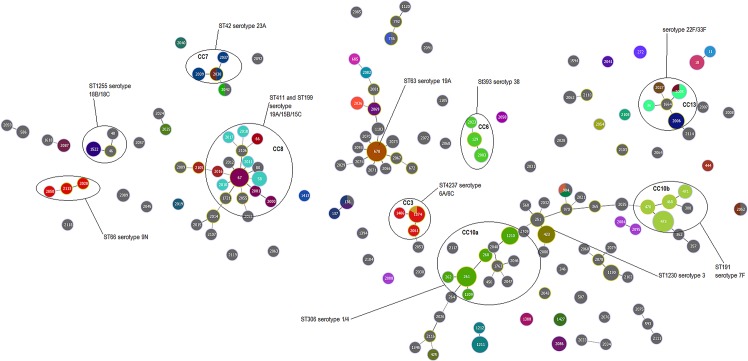
The MLVA1 eBurst diagram depicting invasive *S*. *pneumoniae* isolates from 2007 to 2012 (n = 317). MLST results are overlayed (coloured circles) to allow ease of comparison against MLVA1 results. Grey circles represent isolates that have not been assigned a MLST type. CC are surrounded by a black oval and contain SLV and DLV. The size of the dots corresponds to the number of isolates.

**Fig 3:**
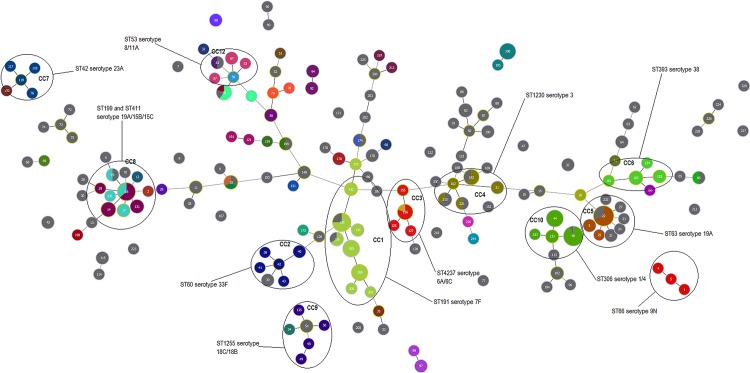
The MLVA2 eBurst diagram depicting invasive S. pneumoniae isolates from 2007 to2012 (n = 317). MLST results are overlayed (coloured circles) to allow ease of comparison against MLVA1 results. Grey circles represent isolates that have not been assigned a MLST type. CC are surrounded by a black oval and contain SLV and DLV. The size of the dots correspond to the number of isolates.

**Fig 4:**
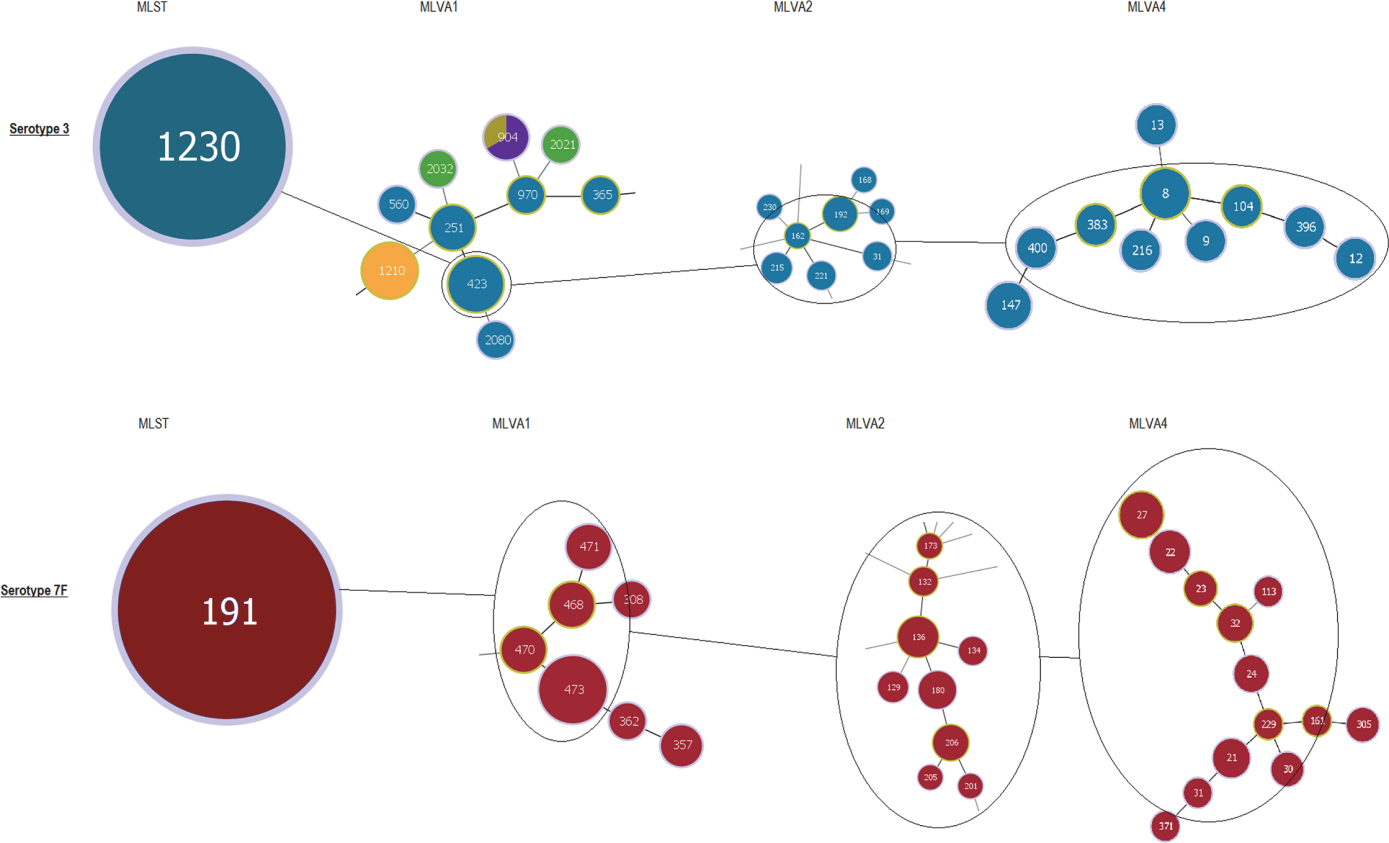
Comparison of discriminatory power for MLST, MLVA1, MLVA2 and MLVA4 when genotyping serotype 3 and serotype 7F in Queensland, Australia. The population structure based on the respective genotypes determined by each genotyping method varies. MLST is considered the least discriminatory as only one genotype is assigned to serotype 3 and serotype 7F, whereas MLVA4 provides increased discrimination by identifying a number of genetically related but different genotypes.

MLST results were also overlayed with the MLVA1 and MLVA2 results. Both MLVA methods are more discriminatory than MLST, as evident by the diversification of MLST types into many MLVA types (e.g. CC10 contains at least five different MLVA1 types with the same MLST type) (Figs. 2 and 3). Interestingly, MLVA1 groups serotypes 1, 3 and 7F into the same cluster (CC10) according to the genotype profile (Fig. 2). Similarly, MLVA2 groups most of the clusters together as isolates appear to have SLV or DLV of another isolates (Fig. 3). However for some serotypes, MLVA4 provides higher discrimination than MLST and the other MLVA methods, evident by the high diversification of serotype 7F and serotype 3 (Fig. 4).

Finally, MLST and MLVA4 used in combination with serotyping can identify potential capsule switching. MLST identifies seven potential capsule switches including serotypes 22F to 33F (ST1012), serotypes 18B to 18C (ST1255), serotypes 15B to 15C (ST199), serotypes 1 to 4 (ST306), serotypes 19A, 15B and 15C (ST411), serotypes 6A to 6C (ST4237) and serotypes 8 to 11A (ST53) (S1 Fig.). On the other hand, MLVA4 only detects two capsule switches including serotypes 15C to 19A (MT59; ST411), and serotypes 1 to 4 (MT36; ST306). The Queensland *S*. *pneumoniae* isolates are listed in the supplementary data (S1 Table) which contains MLVA1, MLVA4, MLST and serotype for comparison.

## Discussion

MLVA has emerged as an alternative genotyping technique to the ‘gold standard’ MLST as it has higher discriminatory power, is fast and inexpensive [20–21, 24]. Results in this study support this finding as MLVA4 is vastly cheaper and faster than MLST, and is still comparable to the costs and laboratory processing time of the other published MLVA methods (Table 2). Reducing time and costs for genotyping will have an impact on the public health field by having the ability to resolve “large and complex outbreak situations” [24].

The choice of the ten VNTRs for our MLVA4 method was based on a Hunter-Gaston Diversity of ≥0.8, as well as an anchor locus with low discrimination to determine long-term changes (BOX-02), and an extra locus with high discriminatory power for specific serotypes (Spneu19). Of the eight highly discriminatory VNTR loci used in previously published MLVA papers, seven VNTRs are included in the modified MLVA4 method [20–23]. MLVA4 maintains high congruence with the other MLVA methods and MLST (Table 2).

When comparing the different genotyping methods, MLVA4 method maintains a high discriminatory power whilst minimising the number of ‘99’, and is significantly more accurate in representing the Queensland pneumococcal population structure than MLST. Clusters of isolates can be observed in more detail and can glean more information when combined with isolate information such as antibiotic resistance or location of disease (not investigated in this study). Admittedly, additional markers have increased the discriminatory power when applied to Queensland, Australian isolates however we have maintained minimal laboratory work to three multiplexes.

MLST was proven to be less discriminatory although it could still maintain as an ideal method for long-term and international epidemiology studies. A less discriminatory protocol is problematic if it does not detect emerging genotypes or outbreaks in a localised area or state. It is evident that MLVA4 is more efficient at discriminating pneumococcal isolates because, for example, MLST type 191 (serotype 7F) actually can be separated into nine different MLVA4 types (Fig. 4).

However, this study confirms the problem of failing to amplify particular loci, resulting in incomplete genotypes. As a result, a number of primers were re-designed in this study in an attempt to successfully amplify the failing loci (including BOX-10, BOX-12, BOX-13 and Spneu19). Elberse *et al*. [40] reported that 24% of their isolates still contained one or more non-amplified BOX loci even after repeated PCR. This study observed that some loci failed to amplify in specific serotypes, for example BOX-06 failed to amplify 75% of Queensland serotype 7F, indicating that primers needed to be redesigned, or VNTR fragments were absent. Serotype 7F (89% isolates) has been associated with a large number of non-amplified BOX-06 genes [40]. Since the primers for MLVA are located in stable *boxA* and *boxC* regions flanking the *boxB* repeats, the difficulty in amplifying the loci is unlikely. Therefore, it is theorised that gene elements are more likely to be lost (or acquired) if there is a higher average number of *boxB* repeats [31]. It is possible that the BOX-06 region is missing from serotype 7F as there are up to eight different BOX-06 fragment lengths. Therefore BOX-06 was not used in MLVA4 due to a high percentage of ‘99’ and a low Hunter-Gaston diversity. Serotype 7F was the second most common serotype (9%) found in Queensland, therefore using higher discriminatory loci was favoured.

Similarly, Spneu19 loci and BOX-13 could not be detected in serotype 3 and 33F isolates, respectively. The failure to amplify Spneu19 in serotype 3 has also been observed, suggesting that serotype 3 lack *pcpA* which codes for a non-essential surface protein involved in cell adhesion [21, 41]. On the other hand, long BOX-13 fragments (>2000bp) have been identified in serotype 33F isolates, possibly accounting for the ‘99’ results since the AB3130 internal size ladder only reaches 1200bp. Large fragments could be explained by the placement of an insertion sequence (IS) element, making the BOX element appear to be larger than 2kb. The presence of IS elements has been described in another MLVA study [42].

Variations in interpreting the population structure of *S*. *pneumoniae* have been observed when using different genotyping protocols. Already potential capsule switches have been observed between a serotype 19A and 15C in CC8 (MT59; ST411), and a serotype 1 and 4 (MT36; ST306) in CC10 in our Queensland population using MLVA4 (S1 Fig.). MLST also identifies these capsule switches, as well as many others which may indicate false capsule switching since MLST is less discriminatory to discern true genetic background of *S*. *pneumoniae* isolates. When examining the international database, some of these capsule switches can be verified, for example ST199 (serotype 19A/15C), ST1012 (serotype 22F/33F) and ST4237 (serotype 6A/6C), however there is no support for capsule switching of the other strains in the international database. This could mean that the capsule switch is relatively new. Alternatively, MLVA4 is too discriminatory and even though true capsule switch occurs, MLVA4 identifies two distinct genotypes therefore the assumption is that no capsule switching has occurred. Further investigation is required to determine whether MLVA4 could fail to detect capsule switches or that MLST is detecting false capsule switches. Since MLVA4 is highly discriminatory, it may enable detection of capsule switching earlier than MLST would. Accurately and quickly detecting relationships between serotypes may have an impact on the selection of serotypes for future vaccine strategies. Furthermore, the ability to examine CC with higher discrimination using MLVA4 can provide insight into which BOX elements are changing. Serotype 7F (MLVA CC1 or ST191) largely diversifies due to BOX-10 and serotype 3 diversifies due to Spneu17. It is unknown what the specific functions of these elements are, however it is known that VNTRs and BOX elements play a role in bacterial competence and virulence and can influence gene expression downstream [30, 43]. VNTR loci with high diversity (e.g. Spneu17) would allow increased discrimination within localised or short-term studies, whereas VNTR loci with low diversity (e.g. BOX-02 and BOX-11) would allow identification of long term changes.

In conclusion, we have developed a MLVA4 method for genotyping invasive *S*. *pneumoniae*. The main advantage of this new method over other MLVA protocols is the ability to achieve complete MLVA profiles for serotypes while also maintaining a highly discriminatory and fast genotyping technique. Loci that failed to amplify were found to be serotype specific, which may indicate that these BOX elements in these serotypes may be variable and have the capacity to transpose. Further research is required to understand the VNTR genetics of these serotypes as VNTRs and BOX loci are thought to play a role in virulence. MLVA4 is also more suitable for genotyping *S*. *pneumoniae* than MLST as a more diverse population can be visualised and allow accurate tracking of strains across the state. MLST may be more suitable for a national study, rather than state. This study, establishes a population structure prior and post 13vPCV introduction in Queensland, and it is expected that future monitoring will comprehensively and accurately depict the changes in the pneumococcal population. The future perspective of MLVA is that it will emerge as a cheap and fast genotyping method for localised and national studies that can still be used in conjunction with the currently traditional and slower serotyping and MLST methods for characterising *S*. *pneumoniae*.

## Supporting Information

S1 Fig Gel electrophoresis of serotype 33F *S*. *pneumoniae* strains. The white box highlights a large >2000bp BOX-13 fragment in this serotype, identified for isolate number 105, 217, 219, 302, 303, 345, 365 and 367. Isolates 15, 150, 218 and 366 contain fragment lengths of 450bp for BOX-13. A negative control (-ve) is included and size ladders are in beginning and end lanes (100bp ladder and 123bp ladder, respectively.
(TIF)

S1 Table Genotyping data results for MLST, MLVA1, MLVA2 and MLVA4 applied to Queensland invasive *S*. *pneumoniae* isolates from 2007 to 2012. (XLSX)
