# Supplementary figures and images for: Modified MLVA for Genotyping Queensland Invasive Streptococcus pneumoniae
Source: PLoS One. 2015 Apr 29;10(4):e0121870. doi: 10.1371/journal.pone.0121870 (PMC4414480; doi:10.1371/journal.pone.0121870)

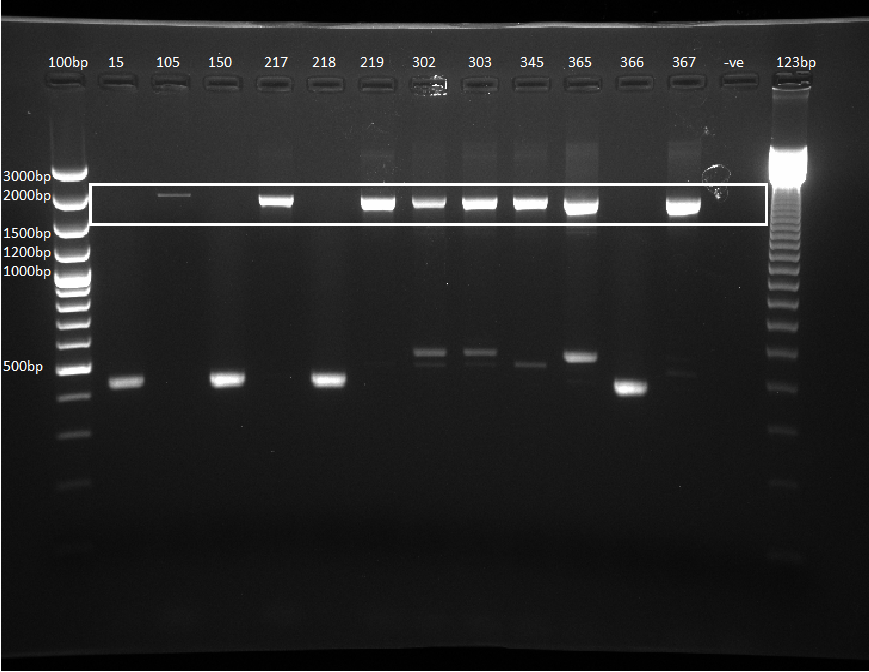

Supplement: S1 Fig — The white box highlights a large >2000bp BOX-13 fragment in this serotype, identified for isolate number 105, 217, 219, 302, 303, 345, 365 and 367. Isolates 15, 150, 218 and 366 contain fragment lengths of 450bp for BOX-13. A negative control (-ve) is included and size ladders are in beginning and end lanes (100bp ladder and 123bp ladder, respectively. (TIF) [file pone.0121870.s001.tif]
